# Electromechanical Coupling Analysis of a Piezoelectric–Flexoelectric–Semiconductor Cantilever Beam

**DOI:** 10.3390/mi17040490

**Published:** 2026-04-17

**Authors:** Yaxuan Su, Xuezhi Wu, Zhidong Zhou

**Affiliations:** 1 Department of Intelligent Manufacturing and Mechanical Engineering, Chengyi College, Jimei University, Xiamen 361021, China; suyaxuan@jmu.edu.cn; 2 Fujian Provincial Key Laboratory of Advanced Materials, College of Materials, Xiamen University, Xiamen 361005, China; wxz20000524@163.com; 3 Xiamen Key Laboratory of Electronic Ceramic Materials and Devices, College of Materials, Xiamen University, Xiamen 361005, China

**Keywords:** flexoelectricity, semiconductor, induced electric potential, electromechanical coupling

## Abstract

This paper presents a theoretical study on the electromechanical coupling response of piezoelectric–flexoelectric–semiconductor (PFS) nanocantilevers by adopting flexoelectric elasticity and semiconductor theory. A unified mechanical–electrical model is established to incorporate a strain gradient, the piezoelectric effect, semiconducting characteristics, and flexoelectricity at micro-/nanoscales. Analytical solutions for deflection, electric potential, and electron concentration are obtained under three types of electrical boundary conditions. Numerical results show that flexoelectricity significantly enhances the effective bending stiffness of the beam under open-circuit conditions with or without surface electrodes, especially in thinner structures. With a fixed external electric potential condition, the applied potential can effectively modulate the deflection by adjusting the polarization field. The induced electric potential, under the open-circuit condition with surface electrodes, exhibits a peak value at a critical thickness and flexoelectric coefficient due to the synergistic effect of the strain gradient and flexoelectricity. The electron screening effect induced by the high doping concentration is found to suppress the induced potential considerably. The present work provides a fundamental understanding of PFS coupling and provides guidance for the design of high-sensitivity micro–nano-electromechanical systems/devices.

## 1. Introduction

Flexoelectricity characterizes the electromechanical coupling between the polarization (or electric field) and the strain gradient in materials, which plays a significant role in the performance of novel micro-/nano-actuators, sensors, and energy harvesters [1,2,3]. Different from traditional piezoelectric devices, flexoelectric-based devices can employ a wider range of dielectric materials and exhibit a significant size effect [4], leading to an evident effective piezoelectric response at the micro-/nanoscales [5]. Mashkevich and Tolpygo [6] first discovered the coupling between polarization and strain gradient. Subsequently, Kogan [7] proposed that a strain gradient can induce polarization in centrosymmetric crystals and introduced the flexoelectric coefficient, which was soon verified experimentally by Bursian and Zaikovskii [8]. Due to the small flexoelectric coefficients of early materials and weak strain gradients in macrostructures, the flexoelectric effect was much weaker than the piezoelectric effect. With the development of new dielectric materials, micro-/nano-structures, and micro-/nano-technologies, the flexoelectric effect in materials has attracted increasing attention. Maranganti [9] first established the flexoelectric constitutive theory for non-centrosymmetric materials within the framework of continuum mechanics, clarifying the inherent correlation between strain gradient and polarization response. Based on molecular dynamics simulations, Majdoub et al. [10] found that the contribution of the flexoelectric effect can exceed that of the traditional piezoelectric effect as the thickness of a nano-cantilever decreases to the nanometer scale, revealing the size-dependent relationship between strain gradient and equivalent piezoelectric coefficient. Shen and Hu [11] established a theoretical framework for the electromechanical coupling of dielectric materials including surface effects, flexoelectric effects, and electrostatic forces by a variational principle. Based on their theory, they analyzed the static electromechanical response [12], vibration and electro-buckling behaviors of flexoelectric nano-beams [13], as well as flexoelectric energy harvesters [14]. Zhou et al. [15] uniformly solved the electromechanical coupling responses of flexoelectric cantilevers under three typical electrical boundary conditions using a variational method based on the electric Gibbs free energy. For the first time, they clarified the relationship between the induced electric potential, flexoelectric coefficient, and structural size under open-circuit electrical conditions. On this basis, they further investigated the electromechanical coupling behaviors of flexoelectric cantilever energy harvesters [16], bilayer flexoelectric–piezoelectric sensors [17], flexoelectric beams on elastic substrates [18], and functionally graded flexoelectric–piezoelectric beam structures [19,20]. Zhang et al. [21] established a model for porous functionally graded piezoelectric nano-beams and analyzed the effects of porosity, applied voltage, flexoelectric effect, and boundary conditions on the static deformation and natural frequency of the beams. Zhou et al. [22] constructed a theoretical model for bilayer circular nano-plates considering both flexoelectric and surface effects and discussed the influence of these two effects on the bending deflection of the plates. Rout and Kapuria [23] proposed a flexoelectric actuator model with both shear lag and peel stress, which couples the flexoelectric and piezoelectric effects and demonstrates the size effect of the actuator at the micro–nanoscales.

Third-generation semiconductors with both piezoelectric and semiconducting effects have been widely employed in novel electronic devices such as flexible electronics, intelligent sensing, and energy harvesting [24,25,26]. Applied mechanical loads can alter the polarization, internal electric potential, and carrier concentration inside the semiconductor. The interaction between multiple physical fields further tunes the intrinsic properties of the semiconductor, thereby realizing the mutual conversion between mechanical and electrical signals. In addition, the flexoelectric effect can also regulate the potential, polarization, and carrier concentration in piezoelectric semiconductors (PSs), thus exerting a significant influence on the performance of piezotronic devices. Navaez et al. [27] experimentally verified that a polarized electric field can be generated in ZnO semiconductor nanowires under bending deformation. Ren et al. [28] introduced a strain gradient field in variable-cross-section structures to tune the performance of PS nanowires and established a one-dimensional model considering the coupled piezoelectric–flexoelectric effect under axial tension. Furthermore, based on Mindlin’s strain gradient theory, approximate analytical solutions for the non-uniform electromechanical field and carrier concentration distribution were obtained. Their results show that carrier perturbation can enhance the flexoelectric effect. Based on the bending model and drift-diffusion theory of semiconductor physics, Qu et al. [29] investigated the motion of carriers dominated by the flexoelectric effect in a PS composite beam. Sun et al. [30] analyzed the electromechanical response of PS composite beams with the flexoelectric effect. Their results indicate that the induced electric potential inside the PS beam exhibits obvious size dependence due to the flexoelectric effect. Zhang and Shen [31] utilized the finite element method to analyze the electromechanical couplings and nonlinear carrier transport in flexoelectric semiconductors based on the fully coupled nonlinear equations. Wei et al. [32] designed a sandwich structure consisting of two piezoelectric layers and one flexible semiconductor layer and established the constitutive equations of the composite structure using piezoelectric and flexoelectric semiconductor theories. The analysis demonstrated that, at the micro–nanoscales, the electric field induced by the piezoelectric effect is weaker than that induced by the flexoelectric effect in the composite beam. Yang et al. [33] proposed a new size-dependent nonlinear model to study the interaction among the flexoelectric effect, piezoelectric effect and semiconducting properties in PS nanofibers. It is found that under a pair of tensile stresses, the flexoelectric effect can enhance the piezoelectric effect, while the semiconducting property weakens the size effect. Most present studies mainly focus on the influences of piezoelectric and flexoelectric effects on the electric field distribution, carrier concentration distribution, and migration inside semiconductor structures. It is of great importance to investigate the effects of flexoelectricity and carrier concentrations on the output electric potential of PFS nanosensors. However, to our knowledge, there are only a few theoretical analyses on the output electric potential of PFS nanosensors.

The objective of the present study is to investigate the electromechanical coupling response of PFS nano-beams under three electrical boundary conditions: open circuit without surface electrodes (OC), closed circuit with a fixed external electric potential (CCF), and open circuit with surface electrodes where an induced electric potential is generated by mechanical deformation (OCI). Based on flexoelectric elasticity and semiconductor theory, the electric Gibbs free energy and the variational principle are adopted to establish the governing equations for nanobeams with the three electrical boundary conditions. Analytical solutions for the deflection, induced electric potential, and electron concentration are derived. The normalized effective deflection and induced electric potential within the beams are analyzed and discussed, with emphasis placed on the effects of flexoelectricity and initial electron concentration on the electromechanical coupling response.

## 2. Analysis of a PFS Beam Under Three Electrical Boundary Conditions

**Figure 1:**
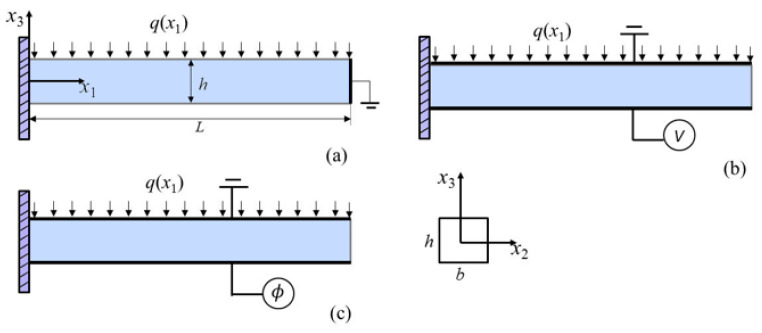
Sketch of the cantilever beams with a distributed lateral force $q(x_{1})$. (**a**) Under the open-circuit condition (OC); (**b**) under the closed-circuit condition with a fixed electric voltage *V* (CCF); and (**c**) under the open-circuit condition with surface electrodes and an induced electric potential ϕ (OCI).

Consider a PFS cantilever beam mechanically fixed at the left end, with different electrical boundary conditions, as shown in Figure 1. The geometric length, width, and thickness of the beam are denoted as *L*, *b*, and *h*, respectively. The OC, CCF, and OCI conditions have been considered, which are depicted in Figure 1, respectively. Under the OC condition, the top and bottom surfaces are charge-free with electric potential set to zero at the right end. Under the CCF condition, the electrodes cover the top and bottom surfaces, which are connected to the ground and an external voltage. Under the OCI condition, the surface electrodes develop an induced electric potential due to mechanical deformation. For PFS materials, the mathematical formulation based on the extended linear theory is used. Here, the electric Gibbs free energy density U for PFS material can be expressed as [34]

(1) $$U=\frac{1}{2}\sigma_{ij}\varepsilon_{ij}+\frac{1}{2}\sigma_{ijk}\varepsilon_{ij,k}-\frac{1}{2}D_{i}E_{i}+\frac{1}{2}J_{i}n_{j},$$

where $\sigma_{ij}$ and $\varepsilon_{ij}$ are the classical Cauchy stress tensor and strain tensor. $\sigma_{ijk}$ and $\varepsilon_{ij,k}$ are the higher-order stress tensor and strain gradient due to flexoelectricity. $E_{i}$ and $D_{i}$ are electric field and electric displacement, respectively. $J_{i}$ and n are the currents of charge carriers and the carrier concentration of semiconductor materials, respectively. The total electrical enthalpy of the solids can be defined as [15]

(2) $$H=∭Udv-∯t_{i}u_{i}ds-∯r_{i}v_{i}ds+∯\varpi \phi ds,$$

where $t_{i}$ and $u_{i}$ are the traction and displacement on the surface, $r_{i}$ and $v_{i}$ are the higher-order traction and normal derivative of displacement on the surface, and ϖ and ϕ are surface charge density and electric potential.

Considering the Bernoulli–Euler beam model, the displacement of the beam can be written as

(3) $$u_{1}=-x_{3}w_{,1},\;\;u_{2}=0,\;\;w=w\left(x_{1}\right),$$

where $u_{1}$, $u_{2}$, and w are the displacements in the $x_{1}$, $x_{2}$, and $x_{3}$ directions, respectively. The corresponding strain and strain gradients are expressed as

(4) $$\varepsilon_{11}=-x_{3}w_{,11},\;\;\varepsilon_{11,3}=-w_{,11},\;\;\varepsilon_{11,1}=-x_{3}w_{,111},$$

The strain gradient $\varepsilon_{11,1}$ could be neglected for a slender beam due to being too small compared to $\varepsilon_{11,3}$. Similarly, the electric filed and electric displacement components in the length direction are negligible. Hence, only the electric filed and electric displacement components in the thickness direction are considered. The constitutive relations of the PFS beam could be express as [15]

(5) $$\left\{\begin{array}{c} \sigma_{11}=c_{11}\varepsilon_{11}-e_{311}E_{3}\\ \sigma_{113}=-\mu_{3113}E_{3}\\ D_{3}=a_{33}E_{3}+e_{311}\varepsilon_{11}+\mu_{3113}\varepsilon_{11,3} \end{array}\right.,$$

where $c_{11}$ is the elastic modulus, $e_{311}$ is the third-rank piezoelectric tensor, $\mu_{3113}$ is the fourth-rank flexoelectricity tensor, and $a_{33}$ is the second-rank dielectric tensor. In the present paper, for the PFS beam, the nonlinear terms of drift currents in the drift-diffusion equations are neglected and the linearized method is used. Here, the hole concentration *p* and electron concentration *n* can be written as $p=p_{0}+∆p$ and $n=n_{0}+∆n$, where $p_{0}$ and $n_{0}$ are the initial concentrations of holes and electrons. ∆p and ∆n are the perturbation concentrations of holes and electrons, respectively. The current equations of the semiconductor beam can be linearized as [28,30,31]

(6) $$\left\{\begin{array}{c} J_{3}^{p}=a_{0}p_{0}\mu_{33}^{p}E_{3}-a_{0}D_{33}^{p}{\Delta p}_{,3}\\ J_{3}^{n}=a_{0}n_{0}\mu_{33}^{n}E_{3}+a_{0}D_{33}^{n}{\Delta n}_{,3} \end{array}\right.,$$

where $J_{3}^{p}$ and $J_{3}^{n}$ are the currents of holes and electrons in the thickness, respectively. $\mu_{33}^{p}$ and $\mu_{33}^{n}$ are the mobility of holes and electrons. $D_{33}^{p}$ and $D_{33}^{n}$ are diffusion constants of holes and electrons, respectively. $a_{0}=1.6\times 10^{-19}C$ is the element charge. For a semiconductor beam, Gauss’s law of electrostatics requires [28,30]

(7) $$D_{3,3}=a_{0}(∆p-∆n).$$

When only the electron concentration is considered in the present paper, the hole concentration could be set to zero. Equation (7) can be simplified to

(8) $$D_{3,3}=-a_{0}∆n.$$

Substituting Equation (5) into Equation (8), we can get

(9) $$a_{33}E_{3,3}+e_{311}\varepsilon_{11,3}=-a_{0}∆n.$$

In the PFS beam, due to the electrically isolated conditions, the electron continuity condition and the electron current densities on two surfaces can be expressed as [28,30,34]

(10) $$\left\{\begin{array}{c} J_{3,3}^{n}=0\\ J_{3}^{n}\left(\pm \frac{h}{2}\right)=0 \end{array}\right..$$

Combining Equations (6) and (10), we obtain

(11) $$n_{0}\mu_{33}^{n}E_{3}+D_{33}^{n}{\Delta n}_{,3}=0.$$

The electric potential Φ is related to the electric field by ${E_{3}=-\Phi }_{,3}$. Integrating Equation (11) and plugging into Equation (9), we can get

(12) $$\Phi_{,33}=\frac{a_{0}n_{0}\mu_{33}^{n}}{a_{33}D_{33}^{n}}\Phi +\frac{{a_{0}C}_{1}}{a_{33}}-\frac{e_{311}w_{,11}}{a_{33}},$$

where $C_{1}$ is an unknown constant. Solving Equation (12), we can get

(13) $$\Phi =C_{2}e^{\alpha x_{3}}+C_{3}e^{-\alpha x_{3}}-\frac{{a_{0}C}_{1}-e_{311}w_{,11}}{\alpha^{2}a_{33}},$$

where $\alpha =\sqrt{\frac{a_{0}n_{0}\mu_{33}^{n}}{a_{33}D_{33}^{n}}}=a_{0}\sqrt{\frac{n_{0}}{a_{33}k_{B}T}}\;$. Here, the Einstein relation [28,35] is used, in which $k_{B}$ is the Boltzmann constant and *T* is the absolute temperature. $C_{2}$ and $C_{3}$ are unknown constants. Therefore, the electric field, electric displacement, stress, and perturbation of electrons can be obtained from Equations (5) and (8).

(14) $$\left\{\begin{array}{c} \sigma_{11}=-c_{11}x_{3}w_{,11}+e_{311}\left(C_{2}{\alpha e}^{\alpha x_{3}}-C_{3}\alpha e^{-\alpha x_{3}}\right)\\ \sigma_{113}=\mu_{3113}\left(C_{2}{\alpha e}^{\alpha x_{3}}-C_{3}\alpha e^{-\alpha x_{3}}\right)\\ E_{3}=C_{3}{\alpha e}^{-\alpha x_{3}}-C_{2}{\alpha e}^{\alpha x_{3}}\\ D_{3}=-a_{33}\left(C_{2}{\alpha e}^{\alpha x_{3}}-C_{3}\alpha e^{-\alpha x_{3}}\right)-e_{311}x_{3}w_{,11}-\mu_{3113}w_{,11}\\ ∆n=\frac{a_{33}\alpha^{2}}{a_{0}}\left(C_{2}e^{\alpha x_{3}}+C_{3}e^{-\alpha x_{3}}\right)+\frac{e_{311}w_{,11}}{a_{0}} \end{array}\right..$$

Substituting Equations (4) and (14) into Equation (1), the electric Gibbs free energy density U for PFS beam can be written as

(15) $$\begin{array}{l} U=\frac{1}{2}c_{11}x_{3}^{2}w_{,11}^{2}-{(e}_{311}x_{3}+\mu_{3113})\left(C_{2}{\alpha e}^{\alpha x_{3}}-C_{3}\alpha e^{-\alpha x_{3}}\right)w_{,11}\;\\ -{\frac{a_{33}}{2}[\left(C_{2}{\alpha e}^{\alpha x_{3}}-C_{3}\alpha e^{-\alpha x_{3}}\right)]}^{2} \end{array},$$

where $J_{3}n_{,3}=0$ is used due to the electrically isolated condition. With regard to the charge neutrality condition in the reference state, ∆n is required to fulfill the global charge neutrality condition of $\iint \Delta ndx_{1}dx_{3}=0$. Let $\Phi \left(\frac{h}{2}\right)=0$ and $\Phi \left(-\frac{h}{2}\right)=\phi (x_{1})$ on the top and bottom surfaces of the PFS beam. The three unknown constants must satisfy the following expressions:

(16) $$\begin{array}{c} C_{2}+C_{3}=\frac{e_{311}h\theta_{0}}{{2a}_{33}L\alpha sinh(\frac{\alpha h}{2})}\\ {C_{2}e}^{\frac{\alpha h}{2}}+C_{3}e^{-\;\frac{\alpha h}{2}}-\frac{{a_{0}C}_{1}-e_{311}w_{,11}}{\alpha^{2}a_{33}}=0\\ C_{2}e^{-\;\frac{\alpha h}{2}}+C_{3}e^{\frac{\alpha h}{2}}-\frac{{a_{0}C}_{1}-e_{311}w_{,11}}{\alpha^{2}a_{33}}=\phi \end{array},$$

where $\theta_{0}=w_{,1}\left(L\right)-w_{,1}\left(0\right)$ denotes the rotation difference between the two ends of the beam. Solving Equation (16), unknown $C_{2}$ and $C_{3}$ could be obtained as follows:

(17) $$C_{2}=\frac{1}{2}(\frac{e_{311}}{a_{33}}\gamma \theta_{0}-\frac{\phi }{2sinh(\frac{\alpha h}{2})}),{\;\;\;C}_{3}=\frac{1}{2}(\frac{e_{311}}{a_{33}}\gamma \theta_{0}+\frac{\phi }{2sinh(\frac{\alpha h}{2})}),$$

where $\gamma =\frac{h}{L\alpha sinh(\frac{\alpha h}{2})}$.

Under the variational principle, δH=0 is required for mechanical and electrostatic equilibrium of the PFS beam. With Equations (15) and (17), we have

(18) $$\delta \int_{v}Udv=\delta \int_{0}^{L}\left[\left(\frac{2sinh(\frac{\alpha h}{2})}{\alpha }-hcosh\left(\frac{\alpha h}{2}\right)\right)\frac{e_{311}^{2}}{a_{33}}\gamma \theta_{0}bw_{,11}+b\mu_{3113}w_{,11}\phi \right]dx_{1}+\;\;\;\;\;\;\;\delta \int_{0}^{L}{\left({\frac{bh^{3}c_{11}}{24}w}_{,11}\right)}^{2}dx_{1}-\delta \int_{0}^{L}\left[\left(\frac{ba_{33}\alpha (sinh(\alpha h)+\alpha h)}{{16sinh}^{2}(\frac{\alpha h}{2})}\right)\phi^{2}\right]dx_{1}.$$

Using the following expressions

(19) δ∫0Lw,112dx1=2∫0Lw,1111δwdx1+2w,11δw,1L0−2w,111δwL0 δ∫0Lϕw,11dx1=∫0Lw,11δϕdx1+∫0Lϕ,11δwdx1+ϕδw,1L0−ϕ,1δwL0 δ∫0Lϕ2dx1=2∫0Lϕδϕdx1.

Equation (18) can be expressed as

(20) $$\begin{array}{l} \delta \int_{v}Udv=[\left(\frac{2sinh(\frac{\alpha h}{2})}{\alpha }-hcosh\left(\frac{\alpha h}{2}\right)\right)\frac{e_{311}^{2}}{a_{33}}\gamma \theta_{0}b+b\mu_{3113}\phi +G_{E}w_{,11}]\delta \left(w_{,1}\right){|}_{0}^{L}\\ -\left(G_{E}c_{11}w_{,111}+b\mu_{3113}\phi_{,1}\right)\delta w{|}_{0}^{L}+\int_{0}^{L}\left(G_{E}w_{,1111}+b\mu_{3113}\phi_{,11}\right)\delta wdx_{1}\\ +\int_{0}^{L}\left[b\mu_{3113}w_{,11}-\left(\frac{ba_{33}\alpha \left(\sinh\left(\alpha h\right)+\alpha h\right)}{{8sinh}^{2}(\frac{\alpha h}{2})}\right)\phi \right]\delta \phi dx_{1} \end{array},$$

where $G_{E}=\frac{bh^{3}}{12}c_{11}$ is the bending rigidity of the beam.

Assume that only a lateral force q($x_{1}$) is applied on the top surface, as shown in Figure 1, yielding $∯t_{i}{\delta u}_{i}ds=\int_{0}^{L}q(x_{1})\delta wdx_{1}$ and $∯r_{i}{\delta v}_{i}ds=0$. When no electrodes are present on the top and bottom surfaces, and left end, the free charge ϖ is zero on these surfaces. At the same time, δϕ=0 at the right end and ϖ=0 at the left end. It follows that ∯ϖδϕds=0 for the OC condition. If electrodes are attached to the top and bottom surfaces with an applied external voltage *V*, then δϕ=0 on both surfaces. In this configuration, both ends are open-circuited with zero free charge ϖ, so that ∯ϖδϕds=0 also holds for the CCF condition. By contrast, for the OCI condition, no external voltage *V* is applied to the electrodes. In the mechanical loading, an electric potential ϕ is induced that is independent of $x_{1}$ but depends on the applied mechanical load. Consequently, ∯ϖδϕds≠0 for the OCI condition. In this section, these electrical boundary conditions are implemented, and analytical solutions are obtained for the bending behavior of PFS nanobeams.

### 2.1. OC Condition

For the OC condition, as discussed above, the variational formulation of the total electrical enthalpy in the PFS beam can be expressed as

(21) $$\begin{array}{l} \delta H_{1}=[\left(\frac{2sinh(\frac{\alpha h}{2})}{\alpha }-hcosh\left(\frac{\alpha h}{2}\right)\right)\frac{e_{311}^{2}}{a_{33}}\gamma \theta_{0}b+b\mu_{3113}\phi +G_{E}w_{,11}]\delta \left(w_{,1}\right){|}_{0}^{L}\\ -\left(G_{E}w_{,111}+b\mu_{3113}\phi_{,1}\right)\delta w{|}_{0}^{L}+\int_{0}^{L}[G_{E}w_{,1111}+b\mu_{3113}\phi_{,11}-q\left(x_{1}\right)]\delta wdx_{1}\\ +\int_{0}^{L}\left[b\mu_{3113}w_{,11}-\frac{ba_{33}\alpha \left(\sinh\left(\alpha h\right)+\alpha h\right)}{{8sinh}^{2}(\frac{\alpha h}{2})}\phi \right]\delta \phi dx_{1}=0 \end{array}.$$

Based on the arbitrariness of δw and δϕ, from Equation (21), the governing equations of the PFS beam can be given as

(22) $$\left\{\begin{array}{c} G_{E}w_{,1111}+b\mu_{3113}\phi_{,11}=q\left(x_{1}\right)\\ \mu_{3113}w_{,11}-\frac{a_{33}\alpha \left(\sinh\left(\alpha h\right)+\alpha h\right)}{{8sinh}^{2}(\frac{\alpha h}{2})}\phi =0 \end{array}\right..$$

For the sake of simplicity, we set $\alpha_{1}=\frac{\alpha \left(\sinh\left(\alpha h\right)+\alpha h\right)}{{8sinh}^{2}(\frac{\alpha h}{2})}$. From Equation (22), the governing equation for the PFS beam can be rewritten as

(23) $$w_{,1111}=\frac{q\left(x_{1}\right)}{G_{D}}=q_{1}\left(x_{1}\right),$$

where $G_{D}=G_{E}+\frac{\mu_{3113}^{2}b}{a_{33}\alpha_{1}}$ is the effective bending rigidity of the PFS beam with the semiconductor and flexoelectric effects. Through Equation (21), the corresponding boundary conditions at the ends of the PFS cantilever beam are

(24) $$\left\{\begin{array}{c} w(0)=w_{,1}(0)=0\\ \frac{e_{311}^{2}}{a_{33}}\alpha_{2}w_{,1}\left(L\right)+G_{D}w_{,11}(L)=w_{,111}\left(L\right)=0 \end{array}\right.,$$

where $\theta_{0}=w_{,1}\left(L\right)$ is used for the cantilever beam in the present paper and

(25) $$\alpha_{2}=\left(\frac{2sinh(\frac{\alpha h}{2})}{\alpha }-hcosh\left(\frac{\alpha h}{2}\right)\right)\gamma b.$$

Solving the governing Equation (23), subjected to a uniformly distributed pressure applied on the top surface, we obtain the deflection of the PFS beam:

(26) $$w_{\left(1\right)}=\frac{q_{1}}{24}x_{1}^{4}-\frac{1}{6}q_{1}Lx_{1}^{3}+\frac{\beta_{1}}{2}{q_{1}L^{2}x}_{1}^{2},$$

where $\beta_{1}=\frac{{3G}_{D}+2\alpha_{2}L\frac{e_{311}^{2}}{a_{33}}\;}{6\left(G_{D}+\alpha_{2}L\frac{e_{311}^{2}}{a_{33}}\right)}\;$.

### 2.2. CCF Condition

When an external voltage *V* is applied to the top and bottom surfaces, it is independent of $x_{1}$. Hence, Equation (21) can be rewritten as

(27) $$\Delta H_{2}=\left[\alpha_{2}\theta_{0}\frac{e_{311}^{2}}{a_{33}}+b\mu_{3113}V+G_{E}w_{,11}\right]\delta \left(w_{,1}\right){|}_{0}^{L}-G_{E}w_{,111}\delta w{|}_{0}^{L}\;+\int_{0}^{L}[G_{E}w_{,1111}-q\left(x_{1}\right)]\delta wdx_{1}=0.$$

The governing equation and corresponding boundary conditions for the PFS cantilever beam can be obtained as

(28) $$\left\{\begin{array}{c} w_{,1111}=\frac{q\left(x_{1}\right)}{G_{E}}=q_{2}\left(x_{1}\right)\\ w(0)=w_{,1}(0)=0\\ {\frac{e_{311}^{2}}{a_{33}}\alpha }_{2}w_{,1}\left(L\right)+G_{E}w_{,11}\left(L\right)+b\mu_{3113}V=w_{,111}\left(L\right)=0 \end{array}\right.,$$

The flexoelectric and semiconductor effects do not affect the governing equation and can induce an effective bending moment via the boundary condition in Equation (28). The corresponding deflection of the PFS beam subjected to a uniformly distributed pressure under the CCF condition can be given as

(29) $$w_{2}=\frac{q_{2}}{24}x_{1}^{4}-\frac{1}{6}q_{2}Lx_{1}^{3}+\frac{\beta_{2}}{2}{{q_{2}L}^{2}x}_{1}^{2},$$

where $\beta_{2}=\frac{3G_{E}+2\alpha_{2}L\frac{e_{311}^{2}}{a_{33}}-\frac{6\mu_{3113}bV}{{q_{2}L}^{2}}}{6\left(G_{E}+\alpha_{2}L\frac{e_{311}^{2}}{a_{33}}\right)}$.

### 2.3. OCI Condition

In this section, the induced electric potential ϕ varies with the mechanical load on the surface. Due to the surface electrodes, the induced electric potential is independent on $x_{1}$. In this case, ∯ϖδϕds≠0. Hence, Equation (21) can be rewritten as

(30) $$\Delta H_{3}=\left[\alpha_{2}\theta_{0}\frac{e_{311}^{2}}{a_{33}}+b\mu_{3113}\phi +G_{E}w_{,11}\right]\delta \left(w_{,1}\right){|}_{0}^{L}-G_{E}w_{,111}\delta w{|}_{0}^{L}\;+\int_{0}^{L}[G_{E}w_{,1111}-q\left(x_{1}\right)]\delta wdx_{1}+b\int_{0}^{L}\left[\mu_{3113}w_{,11}-{a_{33}\alpha }_{1}\phi +\varpi \right]\delta \phi dx_{1}=0.$$

Due to the arbitrariness of δw, the governing equation and corresponding boundary conditions for the PFS cantilever beam can be given as

(31) $$\left\{\begin{array}{c} w_{,1111}=\frac{q\left(x_{1}\right)}{G_{E}}=q_{3}\left(x_{1}\right)\\ w(0)=w_{,1}(0)=0\\ {\frac{e_{311}^{2}}{a_{33}}\alpha }_{2}w_{,1}\left(L\right)+G_{E}w_{,11}\left(L\right)+b\mu_{3113}\phi =w_{,111}\left(L\right)=0 \end{array}\right..$$

It is worth noting that Equations (28) and (31) are highly similar. However, the applied external voltage *V* in Equation (28) is known, whereas the induced electric potential ϕ in Equation (31) is unknown and depends on the mechanical load. An additional condition is required to solve Equation (31). Due to the arbitrariness of δϕ, from Equation (30), the induced electric potential should satisfy the following expression [15]:

(32) $$\int_{0}^{L}(\mu_{3113}w_{,11}-{a_{33}\alpha }_{1}\phi +\varpi )dx_{1}=0.$$

Under the open-circuit condition, no charge is supplied to the electrodes. The induced electric potential can be derived from Equation (32).

(33) $$\phi =\mu_{3113}w_{,1}(L)/a_{33}\alpha_{1}L.$$

Combining Equations (31) and (33), the deflection of the PFS beam subjected to a uniformly distributed pressure under the OCI condition can be obtained:

(34) $$w_{3}=\frac{1}{24}{q_{3}x}_{1}^{4}-\frac{1}{6}q_{3}Lx_{1}^{3}+\frac{\beta_{2}}{2}{{q_{3}L}^{2}x}_{1}^{2},$$

where $\beta_{3}=\frac{3G_{E}+\frac{2e_{311}^{2}}{a_{33}}\alpha_{2}L+\frac{2\mu_{3113}^{2}}{{a_{33}\alpha }_{1}}b}{6\left(G_{E}+\frac{e_{311}^{2}}{a_{33}}\alpha_{2}L+\frac{2\mu_{3113}^{2}}{{a_{33}\alpha }_{1}}b\right)}$. Substituting Equation (34) into Equation (33), we can obtain the analytical expression of the induced electric potential with the mechanical bending as

(35) $$\phi =\frac{\mu_{3113}L^{2}}{a_{33}\alpha_{1}}(\beta_{3}-\frac{1}{3})q_{3}.$$

## 3. Numerical Results and Discussion

In this paper, the electromechanical behavior of the PFS beam is investigated using the formulations presented in Section 2. The beam is assumed to have a slenderness ratio *L/h* = 20 and a width *b* = *h*. n-type ZnO is adopted to explore the flexoelectric effect under various electrical boundary conditions. The flexoelectric coefficient of n-type ZnO is estimated to be on the order of $10^{-7}$ to $10^{-6}$ $C\cdot m^{-1}$. The other material properties for n-type ZnO are taken from [36]: $c_{11}=209.7\;GPa$, $a_{33}=7.889\times 10^{-9}\;C\cdot {\left(V\cdot m\right)}^{-1}$, $e_{311}=-5.1\;C\cdot m^{-2}$, $k_{B}=1.38\times 10^{-23}\;J{\cdot K}^{-1}$, and T=300 K. The initial electron concentration is $n_{0}=10^{23}\;m^{-3}$, and the uniformly distributed transverse pressure applied on the top surface is $q=0.1\;N\cdot m^{-1}$.

**Figure 2:**
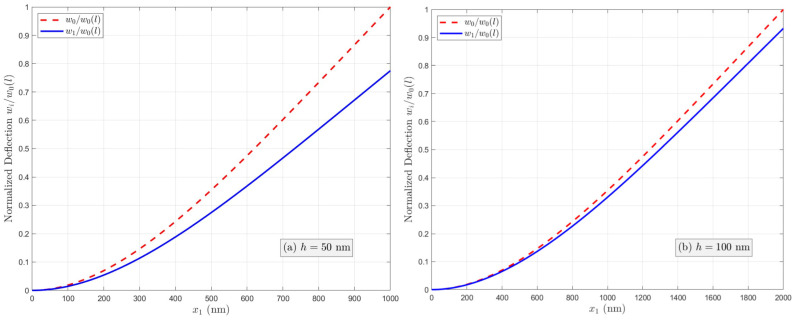
Variation in the normalized deflection under the OC condition when $\;n_{0}=10^{23}/m^{3}$ and ${\mu_{3113}=10}^{-6}$ C/m.

**Figure 3:**
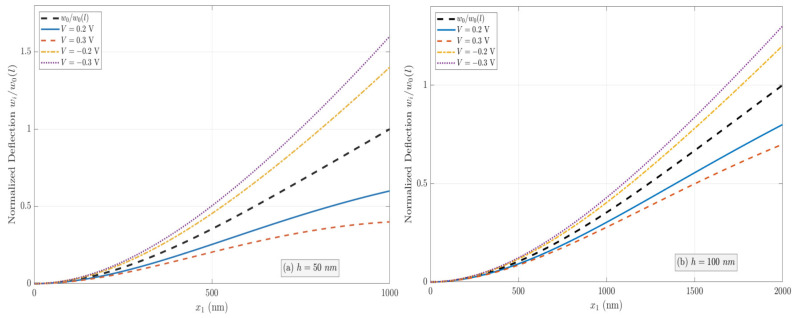
Variation in the normalized deflection under the CCF condition when $\;n_{0}=10^{23}/m^{3}$ and $\mu_{3113}=10^{-6}$ C/m.

**Figure 4:**
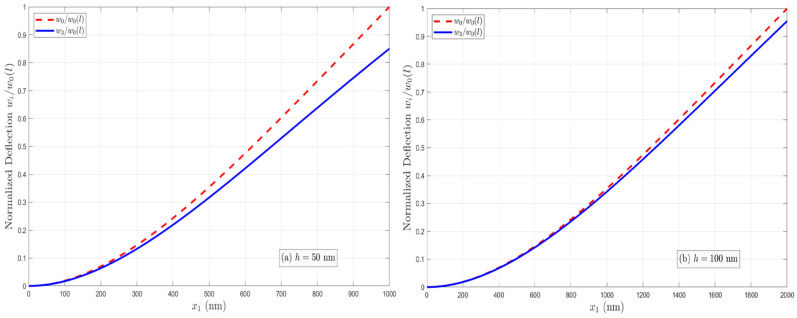
Variation in the normalized deflection under the OCI condition when $\;n_{0}=10^{23}/m^{3}$ and $\mu_{3113}=10^{-6}$ C/m.

As discussed in Section 2, the effective bending rigidities of the PFS nanobeams with three electrical boundary conditions (OC, CCF, and OCI) are different owing to the flexoelectric effect. The normalized effective bending rigidity $G^{\prime }=G_{D}/G_{E}$ of nanobeams increases with decreasing thickness under the OC condition as a result of the coupled flexoelectric and semiconductor effects. However, the apparent bending rigidity under the CCF and OCI conditions depends on the applied electric potential or the induced electric potential. The normalized deflection $w_{\left(i\right)}{/w}_{0}$ (*i* = 1, 2, 3, and $w_{0}=qL^{4}/8G_{E}$ denotes the deflection at the free end in the absence of the flexoelectric and semiconductor effects) of two PFS nanobeams with *h* = 50 nm and 100 nm under three different electrical conditions is plotted in Figure 2, Figure 3 and Figure 4. Figure 2a,b present the normalized deflection of the PFS nanobeam under the OC condition. It is shown that the nanobeam is stiffened by the flexoelectric effect, and the flexoelectric effect becomes weaker with increasing beam thickness. However, the profiles of deflection curves are identical in the presence or absence of the flexoelectric effect. Figure 3a,b illustrate the normalized deflection of the PFS nanobeams subjected to applied electric loads. The normalized deflection can be either smaller or larger when the flexoelectric effect is considered, depending on the direction of the applied electric field. Hence, the nanobeam is stiffer under positive voltage, and softer under negative voltage. However, the normalized deflection will become negative when the external positive electric voltage is larger than a critical value that is a function of the beam thickness. Figure 4a,b show the normalized deflection of the nanobeams associated with the induced electric potential under the OCI condition. Apparently, the converse flexoelectric effect induces a uniform electric field in the beam, which in turn induces a uniform bending moment along the beam that opposes the mechanical load. As a result, the deflection is reduced by the flexoelectric effect. The induced electric potential is thickness-dependent. For a relatively large thickness, the flexoelectric effect is negligible. Decreasing the beam thickness leads to an increasingly significant flexoelectric effect, but the normalized deflection remains positive even as h→0.

**Figure 5:**
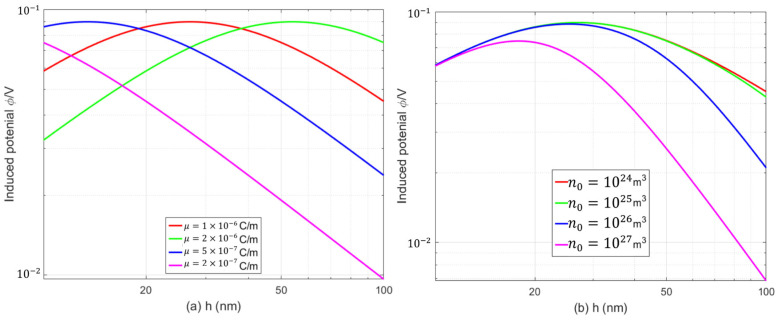
Variation in the induced electric potential with beam thickness: (**a**) various flexoelectric coefficients; (**b**) various initial electron concentrations.

In the design and optimization of PFS devices, it is critical to accurately characterize the internal multi-physics coupling within the structure. For PFS materials, strain gradients can tune the polarization, electric potential, and carrier concentration via the flexoelectric effect, thereby forming a mechanical–electrical–carrier coupling mechanism. Investigating the interactions among these physical quantities is of considerable theoretical and practical significance for material design and device performance optimization. Tuning the flexoelectric coefficient through doping, interface engineering, and other strategies can enhance the response of the polarization field to mechanical deformation, which directly influences the electromechanical conversion performance of semiconductor devices and provides new guidance for the material design of flexible sensors and energy harvesters. In PFS cantilever beams, the electric potential induced by bending deformation can drive carrier migration, thereby enabling the conversion between mechanical and electrical signals. Hence, the induced electric potential in the PFS material plays an important role in flexible sensors and energy harvesters. Equation (35) gives the analytical expression of the induced electric potential with a uniformly distributed pressure. Figure 5 plots the curves of the induced electric potential varying with the beam thickness for the PFS beams corresponding to different flexoelectric coefficients and initial electron concentrations. As observed in Figure 5a, the induced electric potential first increases and then decreases with increasing beam thickness, exhibiting a distinct peak. At a given flexoelectric coefficient, a smaller beam thickness generates a larger strain gradient, thus increasing the induced electric potential. However, when the beam thickness is reduced to a sufficiently small value, the intense induced electric potential will exert a back-action on the nanobeam and suppress structural deformation, which in turn reduces the induced electric potential. As the beam thickness further increases, the constraint imposed by the elastic strain of the material suppresses the development of a large strain gradient, leading to a decrease in the induced electric potential. It can also be seen from Figure 5a that the critical thickness corresponding to the maximum induced electric potential increases with an increasing flexoelectric coefficient of the material, whereas the amplitude of the maximum induced electric potential is independent of the flexoelectric coefficient. Therefore, for materials with a small flexoelectric coefficient, a strong strain gradient field should be achieved by reducing the beam thickness to induce a significant induced electric potential; for materials with a large flexoelectric coefficient, a relatively moderate strain gradient is sufficient to generate a pronounced electric potential output. As shown in Figure 5b, under different initial electron concentrations, the induced electric potential also exhibits a trend of first increasing and then decreasing with the beam thickness, accompanied by a peak potential. In addition, a higher initial electron concentration significantly suppresses the induced electric potential and increases the curvature of the induced electric potential curve. With increasing initial electron concentration, the critical thickness corresponding to the peak induced electric potential decreases, and the peak potential is correspondingly reduced.

**Figure 6:**
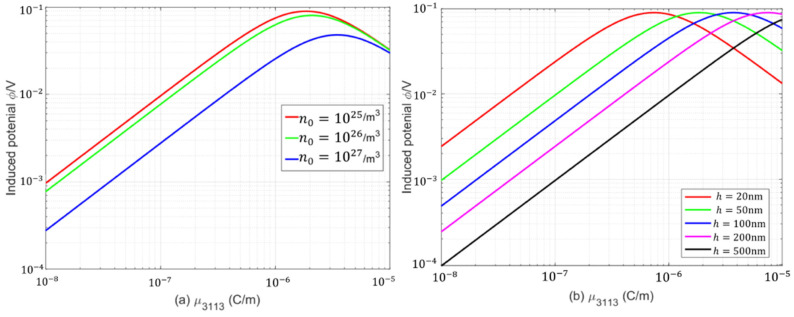
Variation in the induced electric potential with flexoelectric coefficients: (**a**) various initial electron concentrations; (**b**) various beam thicknesses.

Figure 6 presents the evolution of the induced electric potential in a PFS beam as a function of the flexoelectric coefficient, for various initial electron concentrations and beam thicknesses. It can be seen from Figure 6 that the initial electron concentration has a significant effect on the induced electric potential. For different initial electron concentrations, there exists a corresponding critical flexoelectric coefficient at which the induced electric potential attains its maximum value. With increasing initial electron concentration, the induced electric potential of the beam structure decreases, whereas the critical flexoelectric coefficient increases. The results in Figure 6b demonstrate that the induced electric potential first increases and then decreases with the flexoelectric coefficient, exhibiting a peak potential. As the beam thickness increases, the critical flexoelectric coefficient corresponding to the maximum induced electric potential of the system also increases, while the peak potential remains nearly unchanged.

**Figure 7:**
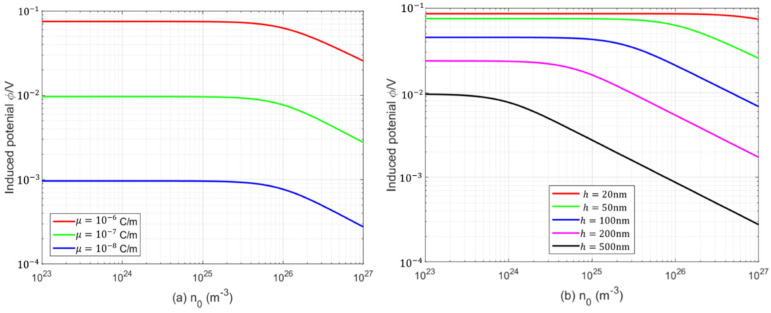
Variation in the induced electric potential with initial electron concentrations: (**a**) various flexoelectric coefficients; (**b**) various beam thicknesses.

Figure 7 illustrates the dependence of the induced electric potential on the initial electron concentration for various flexoelectric coefficients and beam thicknesses. The induced potential remains nearly unchanged at low initial electron concentrations and decreases rapidly once the concentration exceeds a critical value. The flexoelectric coefficient has almost no influence on this critical initial electron concentration, whereas a larger beam thickness results in a lower critical concentration. In addition, the induced electric potential is significantly enhanced by increasing the flexoelectric coefficient or decreasing the beam thickness.

From the above analysis, under the OCI condition, the induced electric potential generated by a mechanical load exerts a reverse influence on the PFS beam. For small sizes or high flexoelectric coefficients, the competition between the polarized electric field and elastic deformation strengthens the electromechanical coupling effect. In contrast, for large sizes or low flexoelectric coefficients, the polarization displays an approximately linear relationship with the flexoelectric coefficient. This behavior stems from the intrinsic impedance characteristics of the material, and the mechanoelectrical conversion efficiency of the system attains a critical state when the flexoelectricity-dominated energy is close to the elastic strain energy.

## 4. Conclusions

Based on flexoelectric elasticity theory and semiconductor theory, this paper systematically investigates the electromechanical coupling behavior of piezoelectric semiconductor nanocantilevers with consideration of the flexoelectric effect. A theoretical model incorporating strain gradient, piezoelectric effect, semiconductor property, and flexoelectric effect is established, which reveals the regulatory mechanisms of flexoelectric and semiconductor effects on the mechanical response and output potential of the structure at micro-/nanoscales. Analytical expressions for the deflection, electric potential, and electron concentration of PFS cantilevers under three typical electrical boundary conditions (OC, CCF, and OCI) are separately derived, and numerical analyses are performed. The results show that under OC and OCI conditions, the flexoelectric effect significantly enhances the effective bending stiffness of PFS beams, and the effective stiffness increases rapidly with decreasing beam thickness. Under the CCF condition, the applied positive voltage suppresses the deflection of the beam, while the negative voltage increases the deflection by weakening the polarization field, which is related to the direction of the mechanical load. Under the OCI condition, there exist a critical beam thickness and a critical flexoelectric coefficient that allow the induced potential to reach its peak value, which is attributed to the synergistic effect between strain gradient and flexoelectricity. In addition, an increase in the initial electron concentration reduces the induced potential, and the electron screening effect also significantly reduces the peak value of the induced potential. Therefore, low-doped semiconductors are suggested for applications in high-sensitivity devices.

## Data Availability

The original contributions presented in this study are included in the article. Further inquiries can be directed to the corresponding author.
